# A framework for digital health equity

**DOI:** 10.1038/s41746-022-00663-0

**Published:** 2022-08-18

**Authors:** Safiya Richardson, Katharine Lawrence, Antoinette M. Schoenthaler, Devin Mann

**Affiliations:** New York University Grossman School of Medicine, New York City, NY USA

**Keywords:** Health policy, Public health

## Abstract

We present a comprehensive Framework for Digital Health Equity, detailing key digital determinants of health (DDoH), to support the work of digital health tool creators in industry, health systems operations, and academia. The rapid digitization of healthcare may widen health disparities if solutions are not developed with these determinants in mind. Our framework builds on the leading health disparities framework, incorporating a digital environment domain. We examine DDoHs at the individual, interpersonal, community, and societal levels, discuss the importance of a root cause, multi-level approach, and offer a pragmatic case study that applies our framework.

## Introduction

Decades of research have identified health differences, based on one or more health outcomes, that adversely affect several defined populations, including rural populations, persons with low incomes, racial and ethnic, and sexual and gender minorities^1^. Early work in the field of health disparities focused on identifying and describing these differences and their potential causes. In the last two decades, there has been a growing understanding of the role of systemic oppression as a root cause of disparities, as well as a commitment to discovering effective interventions^2,3^. The field’s focus on health equity reflects this shift. Health equity refers to the absence of health inequities, differences in health that are unnecessary, avoidable, unfair, and unjust^4^.

As the field of health disparities has matured, we’ve simultaneously witnessed the digital transformation of healthcare. The Health Information Technology for Economic and Clinical Health (HITECH) Act of 2009 sparked the long-awaited adoption of electronic health records (EHRs) by healthcare systems across the country and eventually the development of patient portals, allowing patients online access to key elements of their medical charts. Today, over 95% of hospitals use a government-certified EHR and allow their patients to view health information online^5^. HITECH additionally spurred private industry investment in digital health, including mobile health, wearable devices, remote patient monitoring (RPM), and telehealth, which now is noted in billions per year.

The coronavirus disease 2019 (COVID-19) pandemic highlighted both the continued impact of long-standing systemic oppression on disparate health outcomes as well as the growing importance of digital healthcare. Several studies found significant differences in successful telehealth use in disparity populations^6–8^. Access to digital health is becoming an increasingly important determinant of health. There has been a growing recognition of access as just one of several determinants in the digital environment that impact outcomes^9,10^. These digital determinants of health (DDoH), including access to technological tools, digital literacy, and community infrastructure like broadband internet, likely function independently as barriers to and facilitators of health as well as interact with the social determinants of health (SDoH) to impact outcomes^11,12^.

As digital health becomes increasingly essential, a framework for digital health equity detailing key DDoHs, is needed to support the work of leaders and developers in the industry, health systems operations, and academia. Digital health solution developers include computer scientists, software architects, product managers, and user experience designers. The digital transformation of health requires leaders and developers to understand how digital determinants impact health equity. In this article, we present the Framework for Digital Health Equity, an expansion of the leading health disparities framework. We examine key DDoHs at the individual, interpersonal, community, and societal levels, discuss the importance of a root cause, multi-level approach, and offer a pragmatic case study as an example application of our framework.

## Definitions

### Health disparity populations

The framework applies to all health disparity populations. As defined by the US Office of Management and Budget, these include racial/ethnic minorities, socioeconomically disadvantaged populations, underserved rural populations, and sexual and gender minorities (which include lesbian, gay, bisexual, transgender, and gender-nonbinary or gender-nonconforming individuals). We acknowledge and support the special emphasis placed by the NIMHD on the historical trauma experienced by American Indian groups that were displaced from their traditional lands and African-American populations that continue to endure the legacy of slavery. We additionally include a focus on individuals with disabilities, including those with limitations in their ability to see, see color, hear, etc., that might impact digital accessibility.

### Digital environment

The digital environment is enabled by technology and digital devices, often transmitted over the internet, or other digital means, e.g., mobile phone networks. This includes digital communication, RPM, digital health sensors, telehealth, and the EHR. The digital environment includes elements of the physical/built environment, sociocultural practices, and understanding, as well as the habits and behaviors that dictate how we use these tools. The digital environment exists within and outside of the formal healthcare system.

### Social determinants of health

SDoH are defined by the Centers for Disease Control and Prevention as “conditions in the environments in which people are born, live, learn, work, play, worship, and age that affect a wide range of health, functioning, and quality-of-life outcomes and risks”^13^. These social circumstances are responsible for health inequities as they are heavily shaped by the distribution of money, power, and resources. The SDoH falls into five key categories: healthcare access and quality, education access and quality, social and community context, economic stability and the neighborhood, and the built environment. Determinants in the digital environment, including access, can significantly impact the SDoHs. For example, applications for employment, which influence an individual’s economic stability, are now almost exclusively accessible online.

### Digital determinants of health

The DDoH are conditions in the digital environment that affect a wide range of health, functioning, and quality of life outcomes and risks. The DDoH includes access to technological tools, digital literacy, and community infrastructure like broadband internet and operates at the individual, interpersonal, community, and societal levels. They impact digital health equity, which is equitable access to digital healthcare, equitable outcomes from and experience with digital healthcare, and equity in the design of digital health solutions^10,12^.

## Framework for digital health equity

### National Institute on Minority Health and Health Disparities (NIMHD) Research Framework

The framework for digital health equity is an expansion of the NIMHD Research Framework. This framework, published in 2019, is the culmination of decades of work in the field of health disparities^14^. The framework is organized into several domains, including biological, behavioral, physical/built environment, sociocultural environment, and the healthcare system. It categorizes domains of determinants according to levels of the socioecological model. The SDoH are included primarily in the physical/built environment, sociocultural environment, and the healthcare system domains. The NIMHD Research Framework was an adaptation of the National Institute on Aging (NIA) disparities model, where the healthcare system domain was added because of its particular importance to health. Similarly, because of its critical role in health and healthcare - we incorporate a digital environment domain.

### Framework for digital health equity

**Fig. 1:**
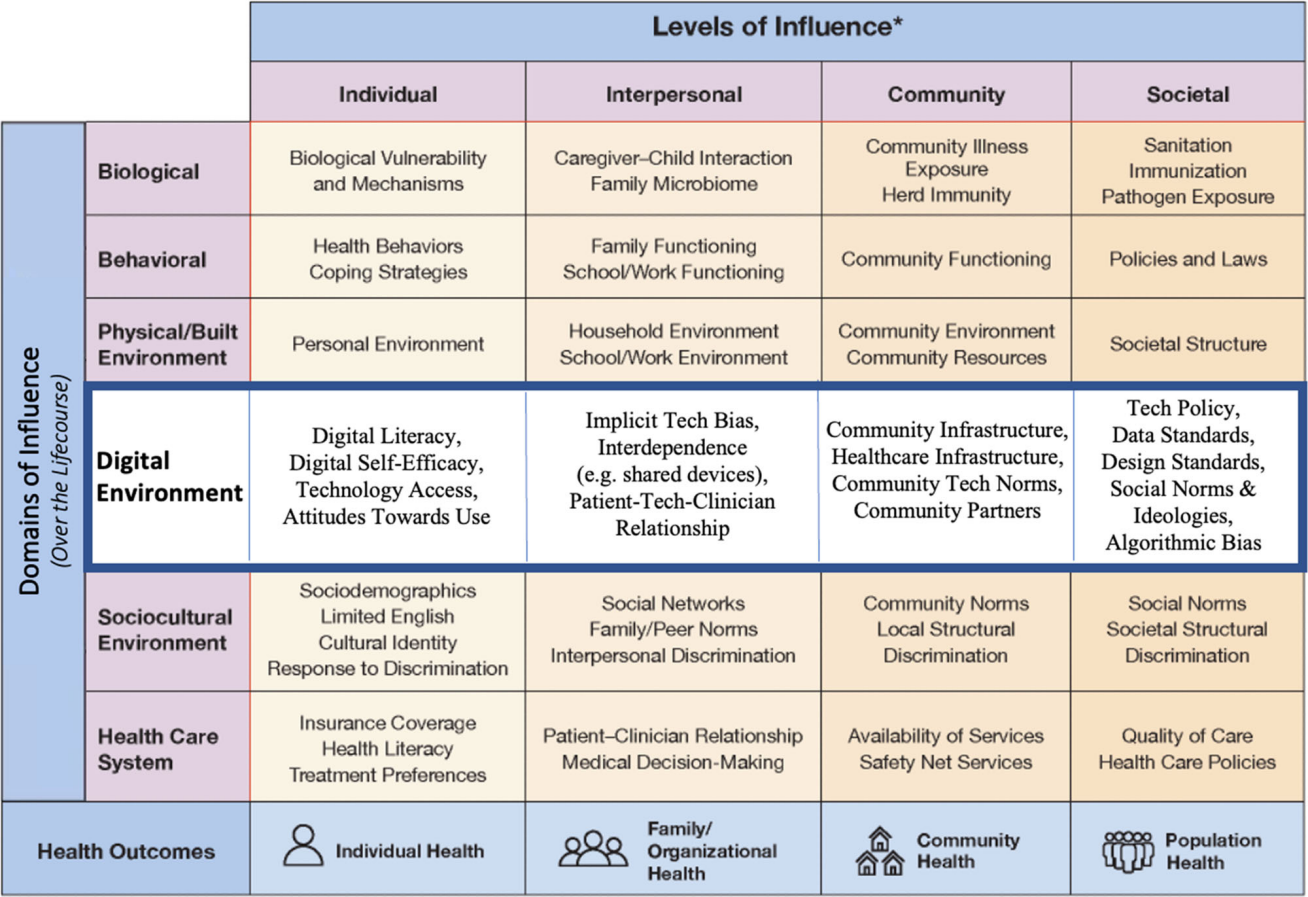
Framework for digital health equity. National Institute on Minority Health and Health Disparities Research Framework Expanded for Digital Health Equity.

The DDoH are incorporated into the NIMHD Research Framework within the digital environment domain (Fig. 1). Determinants are not intended to be exhaustive and often function in ways that are cumulative or interactive.

### Individual-level determinants

Determinants at the individual level include digital literacy, digital self-efficacy, technology access, and attitudes towards use. Digital literacy refers to the skills and abilities necessary for digital access, including an understanding of the language, hardware, and software required to successfully navigate the technology^15^. Self-efficacy is the belief that one can surmount any problem through one’s own effort and is connected to a wide variety of desirable outcomes, including higher performance and achievement striving^16,17^. Digital self-efficacy is an individual’s self-efficacy with regard to the effective and effortless utilization of information technology and predicts proficiency^18^. Digital literacy contributes positively to but does not entirely account for an individual’s sense of digital self-efficacy^19,20^. Others have highlighted similar terms, such as digital confidence, as distinct from digital literacy and instrumental in establishing the digital agency, an individual’s ability to control and adapt to a digital world^20^.

Technology access describes the necessary technological equipment availability to an individual. Attitudes towards use include an individual’s desire and willingness to use, trust in, and beliefs about their ability to use digital tools. Attitudes towards use are adapted from theories of technology adoption such as the technology acceptance model (TAM) and include perceived usefulness and perceived ease of use which predict technology adoption^21^. Trust is also a key construct, as disparity populations can have unique concerns about privacy, security, and surveillance. These concerns can be exacerbated by factors that might be comforting to other groups, for example, affiliation with state or government institutions or the healthcare system. For example, African Americans are more likely than whites to distrust the medical system, report experiencing racism in it, and to express concern about threats to privacy from EHRs^22–24^.

### Interpersonal level determinants

Determinants at the interpersonal level include implicit tech bias, interdependence, and the patient-tech-clinician relationship. These determinants describe relational factors that connect individuals to both digital health technologies and one another. Implicit bias is a term with growing use in the healthcare field, defined as associations outside conscious awareness that lead to a negative evaluation of a person. Implicit Tech Bias is used to describe the impact that unconscious perceptions of an individual’s digital literacy, technology access, and attitudes towards use have on clinician (and affiliated healthcare team members) willingness to enroll and engage individuals with digital healthcare tools. For example, disparity populations have been documented to be less likely to receive invitations to set up patient portal accounts by their clinicians^25^. These clinicians may have been attempting to select patients more likely to successfully use the portal, however implicit bias would have played a role in this assessment and may have contributed to unequal access. Interdependence is used to describe the dependence of two or more people (e.g., family members, caregivers, or friends) on each other for the digital skills, access, and equipment necessary to use digital health tools. For example, low-income households are more likely to share devices and to operate in connection with others^11^. Interdependence can be considered as a positive adaptive mechanism in many contexts, with these bonds serving as positive social capital and facilitating healthy behaviors for both individuals and larger group networks.

The Patient-Tech-Clinician relationship describes the complex interpersonal transformations encouraged by digital technologies, which impact power dynamics between individuals and can help address or exacerbate power imbalances in relationships. For example, the digitization of healthcare may democratize the relationship between the individual and clinician, transforming the paternalistic paradigm of medicine into an equal partnership through data access and transparency^26^. For disparity populations, this has the potential to impact well-documented dimensions of the patient-clinician relationship, including medical mistrust and poor quality communication.

### Community level determinants

Determinants at the community level include community infrastructure, healthcare infrastructure, community tech norms, and community partners. Community infrastructure includes cellular wireless and broadband access, quality, and affordability. Broadband access is considered an important health determinant, access to which should be ensured by the Federal Communications Commission^9^. Digital redlining impacts access to patient portals, RPM, and telehealth^27,28^. Without broadband internet access, patients cannot fully use telehealth in all its forms: asynchronous messaging via patient portals, remote monitoring devices such as blood pressure monitors, or synchronous video connections to consult with a physician.

Healthcare infrastructure includes community access to health systems with advanced digital capabilities, including sophisticated EHR systems, patient portals, and telehealth tools like RPM and simultaneous audio-visual visits. Community tech norms include community preferences for particular tools (i.e., WeChat), high- vs. low-tech solutions, etc. These norms impact health outcomes based on how they compare or contrast with those of the dominant culture and are influenced by a variety of factors, including perceived utility and availability of certain features (e.g., language) which improve acceptability for specific communities. Community partners are an important contribution to the local digital equity ecosystem, the socio-technical systems that work to increase access, and include tech advocacy groups, community health workers, libraries, and digital literacy training programs.

### Societal-level determinants

Determinants at the societal level include tech policy, data and design standards, social norms and ideologies, and algorithmic bias. Tech policy includes the federal, state, and local policies supporting healthcare technology adoption (i.e., HITECH), development and innovation (i.e., 21st Century Cures Act), and security (i.e., HIPAA, Health Insurance Portability and Accountability Act). Data standards are created and maintained by professional organizations, for example, Health Level Seven International produced Health Level Seven (HL7), Fast Healthcare Interoperability Resources (FHIR), and others. The inclusion or exclusion of data relevant to certain populations in these standards impacts the ability of organizations to measure and monitor progress towards equity.

Design standards impact accessibility for those with disabilities and low digital health literacy. For example, Web Content Accessibility Guidelines (WCAG) cover a wide range of recommendations for making Web content more accessible^29^. Recommendations include ensuring a high contrast ratio for colors, that text size can be increased by at least 200%, and not using color alone to convey information. Following these guidelines makes content accessible to a wider range of people with disabilities, including blindness and low vision, deafness and hearing loss, learning disabilities, cognitive limitations, limited movement, speech disabilities, photosensitivity, and combinations of these. Social norms and ideologies are the set of beliefs and philosophies that impact who develops digital tools, what is developed, how it is used, and who it is used by. For example, diffusion of innovation theory is widely adopted in the field and includes the assumption that technology should be developed for early adopters, typically those with excess time and resources, and these tools will eventually trickle down to the general population^30,31^. Other examples include the masculine coding of technology and our assumptions that these tools provide objectivity^32^.

Algorithmic bias includes bias in the use of machine learning and artificial intelligence as well as racial bias in health algorithms that do not use these advanced statistical and computational methodologies. The use of race correction in health algorithms has recently come under scrutiny for potentially contributing to health disparities^33^. For example, the Vaginal Birth After Cesarean Risk Calculator provides a lower estimate of the probability of vaginal birth after prior cesarean for individuals of African-American race or Hispanic ethnicity^34^. The health impact of this may be significant considering that women of color continue to have higher rates of cesarean section, the health benefits of vaginal deliveries are well known, including lower rates of surgical complications, and black women, in particular, have higher rates of maternal mortality^35^. The use of a calculator that lowers the estimate of the success of vaginal birth after cesarean for people of color could exacerbate these disparities.

## An “upstream”, multi-level approach

We hope that the Framework for Digital Health Equity will encourage users and support them in developing an “upstream”, multi-level approach. Health disparities are the result of complex social, environmental, and structural forces. However, interventions in the field have suffered from the Fundamental Attribution Error—overweighting the impact of individual or personal factors and underweighting contextual or situational factors. Health disparities interventions almost always target individual determinants, less often targeting the interpersonal, community, or societal-level determinants^14^. Disparity populations are less likely to benefit from interventions focused on individual-level determinants, as barriers, including limited resources and competing priorities, are greater in these populations^36,37^. Interventions targeting “upstream” determinants at the community and societal levels (i.e., digital infrastructure) are more likely to be effective for these populations^38,39^.

In addition to targeting “upstream” DDoH, a multi-level approach that simultaneously targets the interdependence between the levels of influence can be effective as well. Disparity populations often face structural disadvantages at multiple mutually reinforcing levels^40^. For those studying the effects of such approaches, contemporary methods can provide information about causality at multiple levels as well as interaction effects. These contemporary methods include the sequential, multiple assignments, randomized trial (SMART), multi-level analysis, and the multiphase optimization strategy (MOST) research framework. A multi-level approach allows us to discover the impact of targeting determinants that may be necessary to address to close disparities in outcomes but that are by themselves not sufficient for eliminating disparities.

## Applying the framework for digital health equity: remote patient monitoring use case

The use of RPM, e.g., ambulatory, noninvasive digital technology to capture, and transmit patient data in real-time for care delivery and disease management, is an innovative digital health capability that is rapidly being embedded into our healthcare delivery system. It is increasingly being leveraged for the management of hypertension^41^, diabetes^42^, congestive heart failure^43^, chronic obstructive pulmonary disease^44^, and a range of other chronic conditions^45^. Established non-digital SDOH for RPM uptake include issues such as limited English proficiency (individual level, sociocultural environment domain), disparities in insurance coverage (individual level and healthcare system domain), preferences for social/community-oriented vs. individually driven care (interpersonal level and sociocultural environment domain), device safety and security issues (community level and behavioral domain) and staffing models of medical practices in underserved communities (societal level and healthcare system domain).

Digital health giants and startups now offer an expanding ecosystem of devices, platforms, and products supporting the scale-up of this new technology^46^. Following the usual diffusion of innovation paradigm, many of these offerings are targeting well-resourced settings and populations. However, the dissemination of RPM is early enough that there is an opportunity to use our emerging understanding of the DDoH to alter the usual diffusion curve and build RPM tools that can meaningfully engage health disparity populations. To facilitate this disruption, we highlight RPM digital health equity considerations for the digital health industry, clinical, community, and policy leaders to consider as they grow their RPM products and programs (Table 1).

**Table 1:** Applying the framework for digital health equity: remote patient monitoring use case.

Individual level	Interpersonal level	Community level	Societal level
• Inclusive design of user interface for increased usability for those with low digital literacy. (Digital literacy) • Development of devices/platforms that don’t require wifi and use cellular instead of Bluetooth connectivity. (Access) • Allow patients to actively approve all data transmitted to clinicians. (Interest - Trust)	• Develop an opt-out enrollment process for eligible patients, so enrollment does not depend on clinician referral. (Bias) • Design RPM devices and software to be used by multiple people/devices under one account. (Interdependence) • Include data analysis and interpretation tools in the patient-user interface. (Digital Empowerment)	• Develop products and business models that target safety net health systems as well as academic early adopters. (Health system infrastructure) • Invest in community-based organizations or local partnerships (e.g., libraries) to make devices freely available in underserved communities. (Community partnerships)	• Lobby for Medicare/Medicaid reimbursement for community health workers to support RPM workflows. (Tech Policy) • Engage in HL7 community forums to lobby for the inclusion of SDoH data in FHIR standards. (Data standards) • Require multi-lingual RPM device interfaces. (Design standards*)*

We use RPM as a use case to demonstrate how this framework can be used to highlight opportunities to reshape how digital health is developed, deployed, and disseminated so that diverse communities have an equal opportunity to take advantage of the potential of new digital health technologies.

## Discussion

The rapid digital transformation of healthcare may contribute to increased inequality. Health interventions often lead to intervention-generated inequalities as they are typically adopted unevenly with disparity populations lagging behind^47^. Digital health is particularly vulnerable to this as interventions are likely to disproportionately benefit more advantaged people with greater access to money, power, and knowledge. Digital health leaders and developers in industry, academia, and healthcare operations must be aware of the DDoH and the roles they play to ensure that the use of technology does not widen disparities.

We expand the NIMHD Research Framework to incorporate a digital environment domain detailing key DDoH. Currently, there is no comprehensive framework for digital health equity that addresses determinants at all levels and provides context with the SDoHs. Without an understanding of the DDoHs in context, digital health solution development and research may result in tools and knowledge that are incomplete as they do not address the cumulative or interactive effects of multiple domains. Notably, the framework includes both risk and resilience factors which is key as we support a strengths-based approach to development.

Digital health stakeholders concerned with equity and impact should consider the DDoHs in product development and intervention design and dissemination, incorporating community and societal-level determinants as well as developing multi-level approaches. By expanding the leading health disparities research framework for digital health equity, we hope digital health leaders in the industry, academia, policy, and the community will benefit from decades of progress in the field of health disparities as well as see their work in the larger context of SDoHs so that we might work together towards meaningful progress in using digital means to achieve health equity for all.

## Data Availability

Data sharing not applicable to this article as no datasets were generated or analysed during the current study.
